# Supplementary material for: Influence of Shea tree (Vitellaria paradoxa) on maize and soybean production
Source: PLoS One. 2019 Apr 9;14(4):e0201329. doi: 10.1371/journal.pone.0201329 (PMC6456185; doi:10.1371/journal.pone.0201329)
Supplement: S1 Table — (DOCX) [file pone.0201329.s001.docx]

**Yields Data**

**Maize and Soy bean yield from field experiments**

**Season one maize and soybean field experiment yield**

| **Treatment** | **Replication (2.5m x 2.5m sub plot)** | **Yield (g) per sub plot** | | **Total yield (g) per plot** | | **Total yield (g) per treatment** | |
| --- | --- | --- | --- | --- | --- | --- | --- |
|  |  | **Maize** | **Soy bean** | **Maize** | **Soybean** | **Maize** | **Soy bean** |
| **Mature Shea Treatment** | |  |  |  |  |  |  |
| Shea 1 | 1 | 123 | 225 |  |  |  |  |
|  | 2 | 131 | 237 |  |  |  |  |
|  | 3 | 114 | 253 |  |  |  |  |
|  | 4 | 134 | 251 | **502** | **966** |  |  |
| Shea 2 | 1 | 174 | 283 |  |  |  |  |
|  | 2 | 145 | 252 |  |  |  |  |
|  | 3 | 156 | 220 |  |  |  |  |
|  | 4 | 164 | 234 | **639** | **989** |  |  |
| Shea 3 | 1 | 133 | 221 |  |  |  |  |
|  | 2 | 180 | 232 |  |  |  |  |
|  | 3 | 160 | 245 |  |  |  |  |
|  | 4 | 110 | 253 | **583** | **951** |  |  |
| Shea 4 | 1 | 140 | 233 |  |  |  |  |
|  | 2 | 155 | 260 |  |  |  |  |
|  | 3 | 185 | 284 |  |  | **2.319 kg** | **3.908 kg** |
|  | 4 | 115 | 225 | **595** | **1,002** | **2,319** | **3,908** |
| **Young Shea Treatment** | |  |  |  |  |  |  |
| Young Shea 1 | 1 | 125 | 187 |  |  |  |  |
|  | 2 | 143 | 254 |  |  |  |  |
|  | 3 | 152 | 212 |  |  |  |  |
|  | 4 | 234 | 264 | **654** | **917** |  |  |
| Young Shea 2 | 1 | 268 | 213 |  |  |  |  |
|  | 2 | 254 | 243 |  |  |  |  |
|  | 3 | 231 | 280 |  |  |  |  |
|  | 4 | 259 | 233 | **1,012** | **969** |  |  |
| young Shea 3 | 1 | 315 | 298 |  |  |  |  |
|  | 2 | 286 | 257 |  |  |  |  |
|  | 3 | 294 | 342 |  |  |  |  |
|  | 4 | 265 | 368 | **1,160** | **1,265** |  |  |
| young Shea 4 | 1 | 155 | 253 |  |  |  |  |
|  | 2 | 240 | 340 |  |  |  |  |
|  | 3 | 231 | 374 |  |  | **3.706 kg** | **4.442kg** |
|  | 4 | 254 | 324 | **880** | **1,291** | **3,706** | **4,442** |
| **Control Treatment** | |  |  |  |  |  |  |
| Control 1 | 1 | 550 | 455 |  |  |  |  |
|  | 2 | 580 | 630 |  |  |  |  |
|  | 3 | 660 | 725 |  |  |  |  |
|  | 4 | 720 | 834 | **2,510** | **2,644** |  |  |
| Control 2 | 1 | 575 | 385 |  |  |  |  |
|  | 2 | 645 | 435 |  |  |  |  |
|  | 3 | 550 | 354 |  |  |  |  |
|  | 4 | 540 | 563 | **2,310** | **1,737** |  |  |
| Control 3 | 1 | 653 | 845 |  |  |  |  |
|  | 2 | 720 | 872 |  |  |  |  |
|  | 3 | 672 | 673 |  |  |  |  |
|  | 4 | 725 | 753 | **2,770** | **3,143** |  |  |
| Control 4 | 1 | 562 | 640 |  |  |  |  |
|  | 2 | 728 | 623 |  |  |  |  |
|  | 3 | 654 | 631 |  |  | **10.129 kg** | **10.071 kg** |
|  | 4 | 595 | 653 | **2,539** | **2,547** | **10,129** | **10,071** |

**Season two maize and soybean field experiment yield**

| **Treatment** | **Replication (2.5m x 2.5m sub plot)** | **Yield (g) per sub plot** | | **Total grams per plot** | | **Total yield (g) per treatment** | |
| --- | --- | --- | --- | --- | --- | --- | --- |
|  |  | **Maize** | **Soy bean** | **Maize** | **Soy bean** | **Maize** | **Soy bean** |
| **Mature Shea Treatment** | |  |  |  |  |  |  |
| Shea 1 | 1 | 162 | 252 |  |  |  |  |
|  | 2 | 145 | 274 |  |  |  |  |
|  | 3 | 163 | 263 |  |  |  |  |
|  | 4 | 143 | 276 | **613** | **1,065** |  |  |
| Shea 2 | 1 | 187 | 264 |  |  |  |  |
|  | 2 | 153 | 279 |  |  |  |  |
|  | 3 | 178 | 265 |  |  |  |  |
|  | 4 | 174 | 251 | **692** | **1,059** |  |  |
| Shea 3 | 1 | 153 | 254 |  |  |  |  |
|  | 2 | 186 | 278 |  |  |  |  |
|  | 3 | 183 | 263 |  |  |  |  |
|  | 4 | 194 | 290 | **716** | **1,085** |  |  |
| Shea 4 | 1 | 178 | 245 |  |  |  |  |
|  | 2 | 198 | 298 |  |  |  |  |
|  | 3 | 156 | 239 |  |  |  |  |
|  | 4 | 171 | 296 | **703** | **1,078** | **2,724** | **4,287** |
| **Young Shea Treatment** | |  |  |  |  |  |  |
| young Shea 1 | 1 | 254 | 275 |  |  |  |  |
|  | 2 | 184 | 267 |  |  |  |  |
|  | 3 | 176 | 248 |  |  |  |  |
|  | 4 | 197 | 294 | 811 | 1,084 |  |  |
| young Shea 2 | 1 | 278 | 314 |  |  |  |  |
|  | 2 | 264 | 298 |  |  |  |  |
|  | 3 | 291 | 285 |  |  |  |  |
|  | 4 | 264 | 276 | 1,097 | 1,173 |  |  |
| young Shea 3 | 1 | 245 | 301 |  |  |  |  |
|  | 2 | 258 | 314 |  |  |  |  |
|  | 3 | 232 | 386 |  |  |  |  |
|  | 4 | 293 | 310 | 1,028 | 1,311 |  |  |
| young Shea 4 | 1 | 164 | 243 |  |  |  |  |
|  | 2 | 142 | 276 |  |  |  |  |
|  | 3 | 178 | 321 |  |  |  |  |
|  | 4 | 215 | 348 | 699 | 1,188 | 3,635 | 4,756 |
| **Control Treatment** | |  |  |  |  |  |  |
| Control 1 | 1 | 672 | 547 |  |  |  |  |
|  | 2 | 627 | 637 |  |  |  |  |
|  | 3 | 682 | 763 |  |  |  |  |
|  | 4 | 705 | 825 | 2,686 | 2,772 |  |  |
| Control 2 | 1 | 622 | 742 |  |  |  |  |
|  | 2 | 675 | 537 |  |  |  |  |
|  | 3 | 640 | 581 |  |  |  |  |
|  | 4 | 598 | 598 | 2,535 | 2,458 |  |  |
| Control 3 | 1 | 664 | 803 |  |  |  |  |
|  | 2 | 725 | 875 |  |  |  |  |
|  | 3 | 702 | 736 |  |  |  |  |
|  | 4 | 746 | 858 | 2,837 | 3,272 |  |  |
| Control 4 | 1 | 569 | 653 |  |  |  |  |
|  | 2 | 708 | 664 |  |  |  |  |
|  | 3 | 745 | 679 |  |  |  |  |
|  | 4 | 693 | 705 | 2,715 | 2,701 | 10,773 | 11,203 |
